# Revalidation of the Mentoring Competency Assessment to evaluate skills of research mentors: The MCA-21

**DOI:** 10.1017/cts.2022.381

**Published:** 2022-04-01

**Authors:** So Hee Hyun, Jenna G. Rogers, Stephanie C. House, Christine A. Sorkness, Christine Pfund

**Affiliations:** 1 Institute for Clinical and Translational Research, University of Wisconsin–Madison, Madison, Wisconsin, USA; 2 Wisconsin Center for Education Research, University of Wisconsin–Madison, Madison, Wisconsin, USA

**Keywords:** Research mentor training, Mentoring Competency Assessment, instrument validation, mentoring skills, mentorship common measures

## Abstract

**Introduction:**

The Mentoring Competency Assessment (MCA) is an example of a validated instrument for measuring mentor skills for postsecondary Science, Technology, Engineering, Mathematics, and Medicine research. The purpose of this study was to revalidate the MCA scale using a larger, more diverse population since the original MCA was validated on a small sample of predominantly senior white male faculty.

**Methods:**

The MCA was completed by 1626 mentors from a survey data set of 1759 respondents who participated in eight or more hours of face-to-face *Entering Mentoring*-based training between 2010 and 2019. We conducted principal component analysis (PCA) with varimax rotation to investigate the internal structure of the MCA and Hatcher’s criteria were applied. After a team of mentoring experts independently interpreted the PCA results and reached a consensus on the interpretations of the components, factor analysis and internal consistency reliability analysis were applied to assess the construct validity and the reliability.

**Results:**

While the 26-item MCA instrument was originally validated with six subscales, through the factor and reliability analyses, all the parameter estimates for each item of seven components of 24-item MCA were significant and had relatively high internal consistency; the alpha coefficient for the components ranged from 0.77 to 0.86.

**Conclusions:**

Five items from the MCA have been dropped, leaving a condensed 21 item scale (MCA-21) which loads onto six competencies, and should now be used to effectively measure mentoring skills. We provide recommendations for furthering the scale development and validation of common measures.

## Introduction

Evaluating mentoring effectiveness requires psychometrically sound and reliable measures. Such measures can be used to elucidate the ways in which specific factors, such as mentor knowledge and skills, contribute to the quality and impact of mentoring relationships. These measures can also provide information to mentors to optimize their mentoring practices in ways that contribute most significantly to positive outcomes. Unfortunately, review of the mentorship assessment literature indicates a shortage of measures [1]. A meta-review of mentoring assessment tools in internal medicine between 1990 and 2019 revealed that many “prevailing assessments of mentoring … fail to contend with mentoring’s longitudinal, competency-based, evolving, adapting, entwined, goal-sensitive, context-specific, mentor-, mentee-, mentoring relationship and host organization-dependent nature” [2].

The Mentoring Competency Assessment (MCA) is an example of a validated measure of mentor skills, developed specifically for postsecondary Science, Technology, Engineering, Mathematics, and Medicine research contexts [3]. The MCA was originally developed by a cross-institutional working group to serve as the primary outcome of a multicenter randomized controlled trial (RCT) to assess the effectiveness of the Clinical and Translational Research version of the *Entering Mentoring* curricula [4]. The group proposed 26 items to align with the learning objectives of the six curriculum mentoring competencies : (a) Maintaining Effective Communication (six items), (b) Aligning Expectations (five items), (c) Assessing Understanding (three items), (d) Fostering Independence (five items), (e) Addressing Diversity (two items), and (f) Promoting Professional Development (five items).

The initial validation of the MCA instrument was based on the baseline trial data collected from 283 mentor–mentee pairs across 16 academic health centers, 15 of which were connected to their Clinical and Translational Science Awards (CTSA). The mentors were directed to assess their own skills with all of their mentees; their paired mentees were directed to assess the skills of their mentor in the study. The MCA data were collected via interviews conducted by trained research assistants at each site as part of an expanded protocol. This trial enrolled a relatively homogeneous group of participants in terms of age, career stage, and race. The majority of the mentors were tenured faculty (88%), male (60%), and white (91%); the “average” mentor was a 51-year-old white male full professor in the health sciences with 15 years of mentoring experience. While their mentees were primarily female (58%) and more racially/ethnically diverse, the majority were white (74%) [3,4]. The analysis identified six subscales that were aligned with the six mentoring competencies in the curriculum [3].

In the *Entering Mentoring*-based mentor training RCT, mentors randomized to the training intervention group showed significant gain compared to the control group in their MCA composite score, as well as in all six subscores. Mentees of the trained mentors reported higher MCA scores for these mentors compared to untrained [4]. Since the original publication, the MCA has been widely used as an assessment of mentor training interventions, largely in clinical fields, as well as to assess mentoring programs more broadly [5-7]. The MCA has also been adapted for other contexts and uses, such as a means of assessing the importance of each competency to mentees [7-10]. Researchers have used a version of the MCA as a needs assessment tool and as a mentor self-assessment tool [11]. For example, a study of 135 faculty at an academic health sciences center reported that higher MCA self-assessment ratings were more frequently associated among faculty members with greater academic rank when compared to mentoring experience and clinical/non clinical experience [12].

The purpose of this analysis is to revalidate the MCA scale using a larger, more diverse population beyond senior faculty mentors in academic medical settings. This study revalidates the MCA using a much larger (N = 1626) and more diverse (in terms of participants, institutions, and facilitators) sample. In addition to a different sample, the mentor training curricula to which the MCA was aligned has been refined and expanded beyond the original version utilized in the RCT. This analysis was intended to assess whether the MCA works on a more generalizable population as a measure of mentorship skills.

## Method

### Respondents and Procedure

Research mentor training survey data were collected nationally from 2010 to 2019. This data set included 1759 respondents who participated in an *Entering Mentoring*-based in-person training for 8 h or more to improve their skills as research mentors. The MCA was completed by 1626 mentors after 166 research mentor training events hosted by 54 institutions/organizations. Table 1 shows sample characteristics of the respondents on which the psychometric properties of the measure were tested in this study. The mean age of the respondents was 41.2 years. In the gender distribution, 664 men participated as mentors (41.7%) while 882 women participated (55.4%). The majority of the ethnicity group was non-Hispanic/Latino (n = 1152; 84.2%) and 1056 respondents (67.2%) self-identified as white. Most respondents (n = 889; 50.5%) were faculty. The mean years of experience as a formal research mentor was 5.7 years, and only 297 respondents (20.1%) had participated in a prior mentor training workshop.

**Table 1.** Characteristics of respondents

Characteristics	Mentors (N = 1759)
Mean age in years (range)	41.2 (22–88)
Gender, no. (%)	
Female	882 (55.4)
Male	664 (41.7)
Transgender	2 (0.1)
Other	4 (0.3)
Prefer not to report	41 (2.6)
Ethnicity, no. (%)	
Non-Hispanic or Latino	1152 (84.2)
Hispanic or Latino	144 (10.5)
Prefer not to report	72 (5.3)
Race, no. (%)	
American Indian or Alaskan Native	5 (0.3)
Asian	226 (14.4)
Black or African American	104 (6.6)
White	1056 (67.2)
Other	39 (2.5)
Prefer not to report	83 (5.3)
Multiple Choice	59 (3.8)
Academic rank, no. (%)	
Early career faculty	306 (15.4)
Late career faculty	583 (29.2)
Research staff	189 (9.5)
Graduate student	283 (14.2)
Post-doctoral fellow	178 (8.9)
Other (Lecturer, Dean, Training program director, Administrator, etc.)	455 (22.8)
Years of experience as a formal research mentor, no. (range)	5.7 (0–50)
Prior mentor training workshop participation, no. (%)	
Yes	297 (20.1)
No	1183 (79.9)

### Instrument

The MCA instrument consists of 26 items on a Likert-type scale (1 = Not at all skilled, 4 = moderately skilled, 7 = Extremely skilled). The 26-item MCA served as the primary outcome for the trainings and asked mentors to rate a retrospective pre score and post score for their own skills in mentoring. The post scores were analyzed for the revalidation of the MCA. The MCA was developed and validated by Fleming et al. [3] using the baseline data collected in 2010. Six subscales were originally identified: (a) Maintaining Effective Communication (six items), (b) Aligning Expectations (five items), (c) Assessing Understanding (three items), (d) Fostering Independence (five items), (e) Addressing Diversity (two items), and (f) Promoting Professional Development (five items) [3]. The draft instrument was reviewed by the UW Survey Research Center and tested using both cognitive and standard pilot interviews before being finalized for use in RCT [3].

### Analysis

To investigate the internal structure of the MCA, principal components analysis (PCA) with varimax rotation was applied. PCA is a multivariate technique typically used to reduce a large set of variables to a small set, while containing as much of the information (variation) as possible. Several criteria were applied to determine how many components should be retained to explain most items of the MCA using Hatcher’s criteria [13]:

1. The point of inflexion displayed by the scree plot.
2. The eigenvalues criterion. The eigenvalue-one criterion was considered in conjunction with other criteria (e.g., scree plot and the proportion of variance accounted for) [14]. This analysis used a cutoff on the eigenvalues of 0.65 when deciding how many components to retain and interpret.
3. The “proportion of variation accounted for” criterion. If the designated number of components do not account for at least 50% of the variance, then the analysis is aborted [15].
4. A given component contains variables with significant loadings, a loading of 0.30 being used as the cutoff point.

After determining the number of meaningful components to retain for the MCA, the team of mentoring experts independently interpreted the factors for all relevant results, discussed their interpretations collectively, and reached complete agreement on the interpretations of the factors and their alignment. Finally, confirmatory factor analysis (CFA) and internal consistency reliability analysis were performed to measure the construct validity and the reliability of the MCA with the components which were retained from the PCA. CFA was used to examine the fit of the measurement model and verify the factor structure of a measurement instrument. The use of CFA to conduct the construct validity of hypothesis-based testing instruments can add a level of statistical accuracy and develop condensed forms of an instrument or confirmation of its possible subdomains [16]. The data were analyzed using Stata SE 16.0 for Mac (Stata Corp, College Station, TX, USA).

## Results

### Principal Component Analysis

**Fig. 1.**
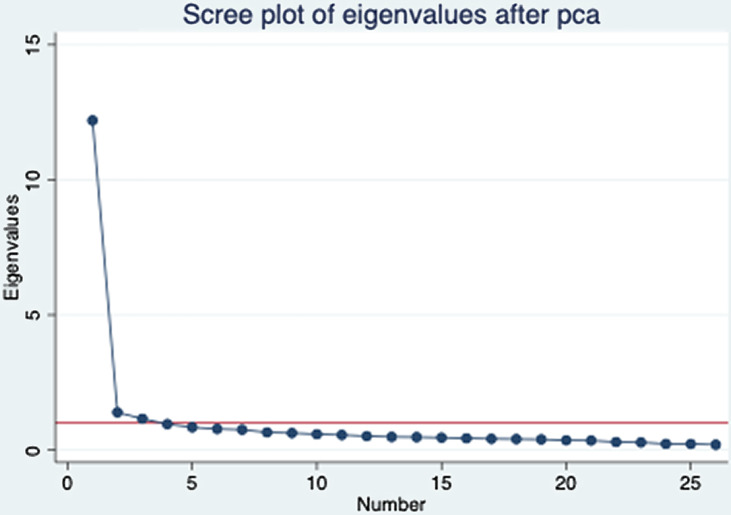
Scree plot of the eigenvalues of the factors. pca = principal component analysis.

The scree plot showed a sharp point of inflection after the first component (Fig. 1). Only three components had initial eigenvalues > 1, with values ranging from 1.16 to 12.20. Since the 26-item MCA was originally validated with six subscales, this study applied a cutoff on the eigenvalues of 0.65 to determine how many components should be retained to explain most items of the MCA. Considering the eigenvalue and the “proportion of variance accounted for” criterion, the first eight components were taken as the starting point for the analysis that explained most items of the MCA with 72% of variation.

Table 2 shows significant component loadings of the eight components with varimax rotation; 24 of the total 26 items were loaded into components. First, in measuring the MCA scale of maintaining effective communication, four of the six items were significantly loaded into one component, (1) active listening, (2) providing constructive feedback, (3) developing a trusting relationship, and (4) accommodating communication style. The item of (5) pursuing strategies to improve communication was not loaded into any components and the item of (6) coordinating with other mentors had high uniqueness which means that the item was loaded itself as a single component.

**Table 2.** Component loadings of the 8 components with varimax rotation (Blank if a loading < 0.30)

No.	Item	Component							
		1	2	3	4	5	6	7	8
Maintaining effective communication									
1	Active listening			0.54					
2	Providing Constructive feedback			0.48					
3	Developing a trusting relationship			0.33					
4	Accommodating communication style			0.54					
5	Pursuing strategies to improve communication								
6	Coordinating with other mentors								0.93
Aligning expectation									
7	Setting clear relationship expectations	0.60							
8	Aligning expectations	0.55							
9	Considering differences may impact expectations						0.30		
10	Setting research goals				0.62				
11	Developing strategies to meet goals				0.64				
Assessing understanding									
12	Assessing mentee knowledge				0.32				
13	Estimating mentee ability	0.36							
14	Enhancing mentee skills	0.31							
Fostering independence									
15	Motivating mentees					0.65			
16	Building confidence					0.64			
17	Simulating creativity							0.31	
18	Acknowledging mentees’ professional contributions							0.49	
19	Negotiating path to independence							0.55	
Addressing diversity									
20	Accounting for biases and prejudices						0.59		
21	Accounting for different backgrounds of mentors and mentees						0.67		
Promoting professional development									
22	Helping network effectively		0.51						
23	Setting career goals		0.46						
24	Helping establish a work/life balance		0.42						
25	Understanding impact as role model								
26	Helping mentees acquire resources		0.51						

Second, items of aligning expectation scale and items of assessing understanding scale were mixed and loaded into three different components. The four items, (7) setting clear relationship expectations, (8) aligning expectations, (13) estimating mentee ability, and (14) enhancing mentee skills, were loaded into one component. Three items, (10) setting research goals, (11) developing strategies to meet goals, and (12) assessing mentee knowledge, were loaded into another component. The item of (9) considering differences that may impact expectations was loaded into the other component with two items of the addressing diversity scale, (20) accounting for biases and prejudices and (21) accounting for different backgrounds of mentors and mentees.

Third, the five items of the fostering independence scale were split up into two different components. The two items, (15) motivating mentees and (16) building confidence, were loaded into one component. Three items, (17) simulating creativity, (18) acknowledging mentees’ professional contributions, and (19) negotiating a path to independence, were loaded into the other component.

Lastly, four of the five items in the promoting professional development scale, (22) helping network effectively, (23) setting career goals, (24) helping establish a work/life balance, and (26) helping mentees acquire resources, were loaded into one component, while the item of (25) understanding impact as role model was not loaded into any components.

### Confirmatory Factor Analysis and Reliability Analysis

Based on the results of the PCA, CFA, and Cronbach’s alpha analysis were performed to measure the construct validity and reliability of the MCA with eight components. In the CFA, we applied maximum likelihood estimation and principal components to assess how well the 24 items measured the eight components and applied the four goodness of fit statistics: chi-square, root mean square of approximation (RMSEA), comparative fit index (CFI), Tucker–Lewis index (TLI), and standardized root mean squared error (SRMR).

Two items, (5) employing strategies to improve communication and (25) understanding impact as role model, were excluded from the factor analysis and Cronbach’s alpha analysis, since these items were not significantly loaded into any components in the PCA. The single item which had high uniqueness, (6) coordinating with other mentors, was not included in the factor analysis and Cronbach’s alpha analysis since one item cannot measure the validity or reliability of a scale, though the item may have practical value.

Table 3 shows standardized factor loadings and Cronbach’s alpha scores for the seven components of 24-item MCA. At a minimum, the following indices should be reported and measured in combination: chi-square; RMSEA; CFI; TLI; and SRMR [17]. A seven-component structure was validated (*χ* ^2^ = 2085.970, *p* < 0.001, RMSEA = 0.079, CFI = 0.905, TLI = 0.886, SRMR = 0.042) and the hypothesized model of the seven components resulted in an acceptable fit to the data: RMSEA < 0.08; CFI > 0.80; TLI > 0.80; and SRMR < 0.05. Considering the sensitivity of the chi-square statistic to sample size, overall goodness-of-fit indices were applied to measure model adequacy. All the parameter estimates for each item were significant, with standardized factor loadings ranging from 0.47 to 1.00. The alpha coefficient for the seven components is from 0.77 to 0.86, suggesting that the items have relatively high internal consistency.

**Table 3.** Factor loadings and Cronbach’s alpha scores for the 24-item Mentoring Competency Assessment

No.	Item	Factor loading	Cronbach’s alpha
Component 1: Aligning expectations			
7	Setting clear relationship expectations	0.86	0.81
8	Aligning expectations	0.88	
13	Estimating mentee ability	0.47	
14	Enhancing mentee skills	0.62	
Component 2: Promoting professional development			
22	Helping network effectively	0.81	0.82
23	Setting career goals	0.84	
24	Helping establish a work/life balance	0.67	
26	Helping mentees acquire resources	0.61	
Component 3: Maintaining effective communication			
1	Active listening	0.69	0.80
2	Providing Constructive feedback	0.75	
3	Developing a trusting relationship	0.70	
4	Accommodating communication style	0.67	
Component 4: Assessing understanding			
10	Setting research goals	0.84	0.79
11	Developing strategies to meet goals	0.82	
12	Assessing mentee knowledge	0.62	
Component 5: Mentee self-efficacy (new competency)			
15	Motivating mentees	1.00	0.86
16	Building confidence	0.76	
Component 6: Addressing diversity			
9	Considering differences may impact expectations	0.65	0.77
20	Accounting for biases and prejudices	0.79	
21	Accounting for different backgrounds of mentors and mentees	0.75	
Component 7: Fostering independence			
17	Simulating creativity	0.74	0.79
18	Acknowledging mentees’ professional contributions	0.76	
19	Negotiating path to independence	0.75	
Component 8: Navigating mentoring networks (new competency)			
6	Coordinating with other mentors	NA	NA

## Discussion

The results of this analysis demonstrate that the structure of the MCA has changed and needs recrafting. The first eight principal components were included to revalidate the MCA scale and identified which items were most strongly correlated with each component. Table 3 demonstrates the new structure of the MCA. Component 1 (Aligning Expectations) was strongly correlated with four items, (7) setting clear relationship expectations, (8) aligning expectations, (13) estimating mentee ability, and (14) enhancing mentee skills. Component 4 (Assessing Understanding) had larger positive associations with three items, (10) setting research goals, (11) developing strategies to meet goals, and (12) assessing mentee knowledge. Interestingly, the items from these two competencies were varied from the original validation structure (some aligning expectation items loading onto assessing understanding here and vice versa). In retrospect, this realignment is not surprising. In order to establish and align appropriate expectations with mentees, it is essential to have a clear understanding of their abilities so that you can enhance their skills. Likewise, in order to establish an appropriate research development plan, you should have a clear understanding of a mentee’s comprehension of key concepts.

Component 2 (Promoting Professional Development) had large positive associations with four items, (22) helping network effectively, (23) setting career goals, (24) helping establish a work/life balance, and (26) helping mentees acquire resources. These items remained the same as the original validation with the exception of the dropped item 25 on this competency (discussed below). Component 3 also held to its original structure, with four items (1) active listening, (2) providing constructive feedback, (3) developing a trusting relationship, and (4) accommodating communication style being strongly associated, and two items (5 and 6) being dropped (further discussed below).

Component 6 (Addressing Diversity) was strongly associated with three items, (9) considering differences may impact expectations, (20) accounting for biases and prejudices, and (21) accounting for different backgrounds of mentors and mentees. While item 9 was originally under aligning expectations, this item now loads under addressing diversity. This is a case where the curriculum content corresponds to a different competency. The authors had always intended to address diversity throughout the curriculum, which is why considering how personal and professional differences may influence expectations is one of the objectives of the Aligning Expectations session. The concept underlying this item does clearly align with addressing diversity.

Component 7 (Fostering Independence) had large positive associations with three items, (17) stimulating creativity, (18) acknowledging mentees’ professional contributions, and (19) negotiating a path to independence. This structure remains the same as the original analyses, though without items (15) motivating mentees and (16) building confidence, as these items strongly correlated with component 5 (Mentee Self-Efficacy). This component was not one of the competencies addressed in the curriculum when the original analysis was conducted, though Promoting Mentee Research Self-Efficacy has since been added as a core mentoring competency. Further, we now understand motivation and confidence to be central to mentee self-efficacy.

Component 8 (Navigating Mentoring Networks) is only correlated with one item, (6) coordinating with other mentors and was not one of the original competencies. This item was originally created to measure the dynamics within mentoring networks as at the time it was becoming more common to work in mentoring teams, and the mentees participating in the trial were in fact required to have multiple mentors as part of their career development awards. We anticipate that comentoring and mentor teams will become more common models and expanded in future curriculum modules.

This analysis also revealed that two items, (5) pursuing strategies to improve communication, and (25) understanding impact as a role model, did not load on any components with no associations with other items. We concluded that these measures are not capturing training content, or rather, not measuring something that was a part of the training. In reflection, curriculum developers agreed that these items are not directly covered in training. Though participants often report gains in these areas, it is likely a result of developing complementary skills. Further, mentors are asked to reflect on the impact of their own practices, which indirectly touches on modeling.

## Future Directions and Recommendations

Based on this analysis to revalidate the MCA, we recommend dropping items 5, 6, 15, 16, and 25 (pursuing strategies to improve communication, coordinating with other mentors, motivating mentees, building confidence, and understanding impact as a role model). A condensed 21 items scale (now referred to as MCA-21) that loads onto 6 competencies should be used; it effectively measures mentoring skills in the competencies of maintaining effective communication, aligning expectations, fostering independence, addressing diversity, promoting professional development, and assessing understanding. Supplementary Table 1 shows the MCA-21.

An important note about the MCA-21 is that while the items have the majority of their variance loading onto the unit-scaled components, some of the variance can also be explained by other factors, thus demonstrating that there are cross-competency gains. This is a consideration for mentor training evaluation as some workshops may not include the full set of modules covering all targeted competencies, but rather just a few. Based upon our findings here, we recommend that the full revised MCA-21 can be used to capture skill gains despite the length or number of modules covered in the training.

The analysis suggests areas for future scale development. For example, our results suggest a new competency focused on helping mentees navigate mentoring networks. In the existing MCA, this competency is only assessed by one item; “coordinating with other mentors.” Another domain of interest is that of role modeling. These domains can be explored more fully by testing additional items built from existing network measures. To accomplish this work, we plan to use a similar approach to measures development and validation around the mentoring competencies of research self-efficacy and providing motivation [18,19].

Finally, the development and validation of measures such as the MCA-21 are important as we move toward the use of common measures across programs. Common measures allow the broader community to identify and examine the factors that matter in workforce development, such as mentorship, across large, diverse groups of mentors and mentees. Such efforts are being pursued by programs such as the National Research Mentoring Network and the CTSAs [20]. A national CTSA T32/TL1 survey has reported mentorship practices of 50 active CTSA hubs [21]. New NCATS Funding Opportunity Announcements have replaced the TL1 funding mechanism with three optional CTSA award components, including an NRSA predoctoral training grant (T32), an NRSA postdoctoral training grant (T32), and a Research Education Grants Program (R25). To be responsive to both training program faculty requirements and scored review criteria, CTSA programs must strengthen mentorship through the use of evidence-informed mentoring practices, evidence-informed mentor training, assessment of mentoring skills and behaviors, and monitoring of mentorship behaviors [21]. The common measure is being used widely and the MCA-21 described here could serve as an updated, validated, common measure.

## Supplementary material

For supplementary material accompanying this paper visit https://doi.org/10.1017/cts.2022.381. click here to view supplementary material
