# Re-validation of the mentoring competency assessment to evaluate skills of research mentors: The MCA-21 – CORRIGENDUM

**DOI:** 10.1017/cts.2024.637

**Published:** 2024-11-05

**Authors:** So Hee Hyun, Jenna G. Rogers, Stephanie C. House, Christine A. Sorkness, Christine Pfund

There are errors present in the published article. In table 2, table 3 and the fourth paragraph of the Principal Component Analysis Results section on page 3, “(17) simulating creativity” should be corrected to “(17) stimulating creativity.”

The authors apologize for this error.
