# Supplementary material for: Revalidation of the Mentoring Competency Assessment to evaluate skills of research mentors: The MCA-21
Source: J Clin Transl Sci. 2022 Apr 1;6(1):e46. doi: 10.1017/cts.2022.381 (PMC9108003; doi:10.1017/cts.2022.381)
Supplement: Supplementary file 1 [file S2059866122003818sup001.docx]

**Supplementary table 1**. MCA-21 (Based on 21 items)

| **No.** | **Item^[[1]](#footnote-1)^** |
| --- | --- |
| **Maintaining effective communication** | |
| 1 | Active listening |
| 2 | Providing constructive feedback |
| 3 | Establishing a relationship based on trust |
| 4 | Identifying and accommodation different communication styles |
| **Aligning Expectations** | |
| 7 | Working with mentees to set clear expectations of the mentoring relationship |
| 8 | Aligning your expectations with your mentees’ |
| 13 | Accurately estimating your mentees’ ability to conduct research |
| 14 | Employing strategies to enhance your mentees’ knowledge and abilities |
| **Assessing understanding** | |
| 10 | Working with mentees to set research goals |
| 11 | Helping mentees develop strategies to meet goals |
| 12 | Accurately estimating your mentees’ level of scientific knowledge |
| **Fostering Independence** | |
| 17 | Simulating your mentees’ creativity |
| 18 | Acknowledging your mentees’ professional contributions |
| 19 | Negotiating a path to professional independence with your mentees |
| **Addressing Diversity** | |
| 9 | Considering how personal and professional differences may impact expectations |
| 20 | Taking into account the biases and prejudices you bring to the mentor/mentee relationship |
| 21 | Working effectively with mentees whose personal background is different from your own (age, race, gender, class, region, culture, religion, family compositions etc.) |
| **Promoting Professional Development** | |
| 22 | Helping your mentees network effectively |
| 23 | Helping your mentees set career goals |
| 24 | Helping your mentees balance work with their personal life |
| 26 | Helping your mentees acquire resources |

1. Using seven-point Likert scale (1=not at all skilled, 2, 3, 4=moderately skilled, 5, 6, 7=extremely skilled) and asking mentors to rate a retrospective pre score and post score for their own skills in mentoring. [↑](#footnote-ref-1)
